# What do suicide loss survivors think of physician-assisted suicide: a comparative analysis of suicide loss survivors and the general population in Germany

**DOI:** 10.1186/s12910-024-01099-9

**Published:** 2024-09-19

**Authors:** Laura Hofmann, Louisa Spieß, Birgit Wagner

**Affiliations:** Department of Clinical Psychology, Medical School Berlin, Rüdesheimer Straße 50, 14197 Berlin, Germany

**Keywords:** Medical-assisted dying, Assisted suicide, Relatives, Grief, Bereavement, Suicide bereavement

## Abstract

**Background:**

Physician-assisted suicide (PAS) and voluntary euthanasia remain highly debated topics in society, drawing attention due to their ethical, legal, and emotional complexities. Within this debate, the loss of a loved one through suicide may shape the attitudes of survivors, resulting in more or less favorable attitudes towards this topic.

**Aims:**

This study aims to explore and compare the attitudes towards PAS and voluntary euthanasia in a population of suicide loss survivors and the general population, while also considering socio-demographic factors.

**Methods:**

A total of 529 participants, 168 of whom were survivors of suicide loss, completed an online questionnaire on their attitudes (NOBAS) and opinions (open response format) towards PAS and voluntary euthanasia, as well as regarding their legalization in Germany. The analysis consisted of both quantitative and qualitative components.

**Results:**

The entire sample showed positive attitudes towards PAS and voluntary euthanasia in terminally ill persons. Participants were more divided in their attitudes towards PAS in the case of a mental health disorder. Individuals without experienced suicide loss were more liberal regarding legalization in Germany and were more likely to understand the wish for PAS. Survivors of suicide loss were mainly concerned about the consequences for relatives. However, differences between both groups are small.

**Discussion:**

The experience of a loss by suicide influences attitudes towards PAS and voluntary euthanasia. Both groups showed an accepting attitude towards PAS and voluntary euthanasia, but also expressed concerns and fears regarding easy accessibility and consequences for grieving relatives.

**Supplementary Information:**

The online version contains supplementary material available at 10.1186/s12910-024-01099-9.

## Introduction

Physician-assisted suicide (PAS) is now legal in an ever-increasing number of countries, bringing this topic to the focus of social and legal debates. PAS is defined as providing medication with the intention that it will result in the patient’s death [1]. The patient must take this medication independently, in contrast to voluntary euthanasia, where the doctor is authorized to administer the lethal medication. Physician-assisted suicide has been permitted in Germany since the decision of the Federal Constitutional Court in February 2020. Doctors and right-to-die organizations are permitted to provide PAS, however, there are ongoing debates about a new regulation. PAS is currently permitted for all individuals who meet certain requirements. These include, for example, that the individual must be capable of making an informed, free, and conscious decision. However, it is expected that there will be stricter regulations for PAS in the future. Voluntary euthanasia, on the other hand, remains prohibited in Germany.

In recent decades, the discourse surrounding end-of-life choices has become increasingly complex, marked by ethical, moral, and societal considerations. In Germany, the term *(physician-) assisted suicide* is still predominantly used, while the term *assisted dying* is increasingly used internationally as a collective term for both PAS and voluntary euthanasia [2].

Opinions differ widely on PAS and voluntary euthanasia. While some see these methods as long-needed options for self-determination, others see the danger of them becoming too easily accessible [2]. Recent studies have focused on attitudes towards PAS and voluntary euthanasia in the general population while also considering socio-demographic factors [3–5]. For example, a Norwegian study with 3.050 general population participants showed positive attitudes towards the legalization of PAS and voluntary euthanasia for patients with terminal illnesses, but participants were more critical towards PAS for people with mental health disorders or people without illnesses who are tired of life [4]. Younger and non-religious participants were more liberal towards PAS. A recent review from [2], consisting of 21 studies, found that younger age, higher education, higher socio-economic status, and lower religiosity are the most stable predictors of a liberal attitude towards PAS and voluntary euthanasia. Religious beliefs particularly seem to be an integral component of attitudes towards assisted dying. In a study from Belgium, the authors focused exclusively on the attitudes of the Muslim community towards PAS and voluntary euthanasia [3]. Their results indicated a clear rejection of both PAS and voluntary euthanasia, regardless of age and level of education. In a study from New Zealand, individuals from the general population also showed a largely positive attitude towards the topic [5]. The authors found no effect of age, but a moderate effect of religious belief. These studies have already provided insight into the public’s attitudes towards PAS and voluntary euthanasia and show that these attitudes are characterized by various socio-demographic factors in some countries or communities.

Other studies on this topic have focused more intensively on the attitudes of doctors and nursing staff who may be involved in preparing and providing assisted dying services [6–9]. In some studies, rather ambivalent attitudes towards PAS and voluntary euthanasia could be found across medical populations [10]. As doctors are the individuals responsible for providing medical aid in dying, their perspective is not only about legalization per se, but also about their role in the process and the potential conflict that arises against the background of also wishing to cure the patient. In a study from Norway [11], where PAS and voluntary euthanasia are currently not permitted, doctors were generally more opposed to legalization.

So far, most studies focus primarily on the attitudes of the general population and the associated legislation, as well as on medical staff, such as doctors and nurses [4, 11, 12]. However, perspectives shaped by a person’s own lived experience of suicide bereavement are still missing. Therefore, there is still little knowledge if this experience makes individuals more or less liberal in their perception of legalization of PAS. There are several reasons why suicide loss survivors might take a more liberal stance regarding assisted dying. A suicide is a violent and sudden death, which often has long-term and far-reaching consequences for the bereaved [13–15]. In particular, the idea of how the person might have died by suicide (e.g., railway suicides, jumping from high buildings) or even finding the person post-suicide can be highly distressing experiences, leaving the bereaved with long-term negative mental imageries [16]. Therefore, bereaved individuals might perceive PAS as a more peaceful and less violent way to end one's life. Assisted dying also offers the opportunity to say goodbye and accompany the person in their final days. This is something that relatives are unable to do after a suicide and which many experience as immensely difficult to process [17]. The wish for PAS is sometimes communicated by relatives and is less associated with feelings of guilt and responsibility for the death of loved ones [18]. Based on this experience, surviving relatives might take a more liberal view of the legalization of PAS and voluntary euthanasia after a suicide, as they might consider the loss through these methods to be less stressful than a loss through suicide.

However, the suicide bereaved might also show a less liberal attitude towards PAS. Survivors have experienced what it is like to lose a loved one first-hand and are aware of the impact it can have on the bereaved [19]. In some cases, the deceased person may have suffered from a mental health disorder and relatives may be concerned that people with mental health disorders will have access to PAS too quickly. They may also have experienced that the desire for suicide can fluctuate and is sometimes not stable over time.

To the best of our knowledge, no study to date focuses specifically on the attitudes towards PAS within a population of survivors of suicide loss. The purpose of this article is to present and compare the attitudes and thoughts of both survivors of suicide and individuals who have not experienced a loss by suicide. More specifically, the study aims to investigate a) the attitudes of suicide loss survivors and the general population, b) if the two groups differ in terms of their attitudes, c) whether attitudes vary according to socio-demographic factors and d) which topics are considered relevant regarding PAS. In order to be able to categorize the attitudes of bereaved individuals, these are compared with the attitudes of individuals who have not experienced a loss through suicide.

## Methods

### Design and study population

The study followed a cross-sectional design wherein participants filled in an online questionnaire. Participants had to meet the following inclusion criteria for participation: (1) aged 18 years or older, (2) possessed sufficient knowledge of German, and (3) provided signed informed consent. We included individuals with and without suicide loss, in order to compare the attitudes of both groups towards PAS. Individuals were excluded if they lost someone through PAS. Recruitment primarily took place via social media (Facebook, X, Instagram), e-mail mailing lists of various universities and through the Association for the Suicide Bereaved in Germany (AGUS e.V.). The Ethics Committee of the Medical School Berlin approved the study on July 12, 2023, in compliance with the current version of the Declaration of Helsinki (reference number: MSB-2023/117).

### Sample characteristics

A total of 2.047 people accessed the questionnaire, 562 of whom completed it. Of these, 170 were survivors of suicide loss, of which two were excluded due to loss through PAS. Of the 392 participants without a suicide loss, 26 also had to be excluded due to a loss through PAS. A further five participants were excluded due to missing data. This resulted in a total sample of *N* = 529, which was comprised of *n* = 361 individuals without and *n* = 168 with loss by suicide. In both samples, most participants were female with 91.1% in the suicide loss survivor sample and 94.2% in the non-loss sample. The mean age was 46.79 (*SD* = 11.29) years and 43.80 (*SD* = 10.59) years, respectively. All sample characteristics can be seen in Table 1. We did not find significant differences between the groups for the characteristics we collected.

**Table 1:** Sample characteristics (*N* = 529)

	**Suicide Loss (*n* = 168)**	**No Suicide Loss (*n* = 361)**
**Age**	45.79 (11.29), 22–71	43.80 (10.59), 19–75
**Gender (female)**	153 (91.1%)	340 (94.2%)
**Marital status**		
Single	26 (15.5%)	58 (16.1%)
In a relationship	28 (16.7%)	62 (17.2%)
Married	68 (40.5%)	198 (54.8%)
Divorced	10 (11.9%)	35 (9.7%)
Widowed	26 (15.5%)	8 (2.2)
**Level of education**		
Secondary	50 (29.7%)	82 (22.7%)
Upper Secondary	64 (38.1%)	143 (39.6%)
Academic	54 (32.2%)	135 (37.4%)
Other	-	1 (0.3%)
**Religious beliefs**		
None	91 (54.2%)	165 (45.7%)
Christian – Protestant	38 (22.6%)	95 (26.3%)
Christian—Catholic	27 (16.1%)	92 (25.5%)
Muslim	-	1 (0.3%)
Buddhism	7 (4.2%)	5 (1.4%)
Other	5 (3.0)	3 (0.8%)
**Time since suicide loss in years**	7.46 (9.15)	-
**Kinship of deceased**		
Parent	35 (19.9%)	-
Child	28 (16.7%)	-
Sibling	32 (19.0%)	-
Partner	36 (21.4%)	-
Others	37 (22.0%)	-

### Measures

#### Socio-demographic data

Relevant socio-demographic data of the participants were collected, as well as information on the suicide loss and the deceased person, if applicable.

#### Questionnaire on attitudes of the Norwegian Bioethics Attitude Survey (NOBAS)

To assess participants’ attitudes towards PAS and voluntary euthanasia, the questionnaire from the NOBAS was used [4, 20]. The questionnaire consists of eight items that cover various aspects of legalization of PAS and voluntary euthanasia. Opinions are rated on a 5-point Likert Scale from 1 = *strongly disagree* to 5 = *strongly agree.* The analysis is only possible on a descriptive level. While the original questionnaire uses the term *active aid in dying* in most questions, we used the term *assisted suicide,* as this is the commonly used term in Germany. The questionnaire also provides a definition of PAS and voluntary euthanasia in the introduction.

#### Questionnaire on legalization of PAS in Germany

To assess the opinions regarding the legalization of PAS in Germany as well as the personal opinions of the participant, a further eight items were added (e.g., "I would make use of assisted suicide if I were suffering from a serious physical illness"). The items are also rated on a 5-point Likert Scale from 1 = *strongly disagree* to 5 = *strongly agree.* In addition, participants were asked to write down their thoughts and views on PAS in an open response format (“Please let us know your thoughts and opinions on assisted suicide here.”). The term *assisted suicide* was used for these items as well. The questionnaire was developed for this study and can be found in Table S1 in the Supplementary Material.

### Statistical analyses

Analyses were carried out using SPSS Version 28 [21]. Means and standard deviations were calculated for continuous variables, frequencies, and descriptive statistics for categorical variables. T-tests were used to analyze differences in attitudes towards PAS between groups with and without suicide loss. Differences in opinions between different socio-demographic groups were analyzed with several MANOVAs. The qualitative data of the open response format was analyzed following a deductive-inductive approach [22]. The data was first prepared for analysis and codes we subsequently generated, which were then applied to the data. If existing codes did not appear suitable, new codes were generated and applied to the data again. This procedure was repeated until the entire data set had been coded. The coding was carried out with MAXQDA 2022 [23] by two independent researchers (LH, LS) in order to ensure reliability. Any discrepancies were discussed.

## Results

### *Attitudes on PAS and voluntary euthanasia (NOBAS* + *additional items)*

Overall, all participants showed a positive attitude towards PAS (Q1) and voluntary euthanasia (Q2) for people with terminal illnesses and a predominantly positive attitude towards PAS for people with incurable chronic illnesses (Q3). Both groups of participants showed divided attitudes towards PAS for people with mental health disorders (Q4) and for people without any illness but who are tired of living (Q5). Both groups neither agreed nor disagreed with these statements. The participants also mostly approved of the legalization of PAS in Germany (Q9) and were less concerned about the financial enrichment of right-to-die organizations (Q10). Participants in both groups generally showed a high level of understanding regarding the wish for PAS (Q12) and did not tend to be concerned that those affected would choose PAS too quickly (Q13). Participants reported that they would also be more likely to make use of PAS themselves if they were suffering from a severe physical illness (Q11). However, participants in both groups were undecided about PAS in the case of an own mental health disorder (Q16). All results are shown in Table 2.

**Table 2:** Attitudes towards PAS and voluntary euthanasia (NOBAS & own items) (*N* = 529)

	**Strongly disagree**		**Disagree to some extent**		**Neither agree nor disagree**		**Agree to some extent**		**Strongly agree**	
	**SL**	**NSL**	**SL**	**NSL**	**SL**	**NSL**	**SL**	**NSL**	**SL**	**NSL**
Q1: PAS in terminally ill patients with a short remaining life expectancy	5 (3.0%)	3 (0.8%)	5 (3.0%)	9 (2.5%)	5 (3.0%)	5 (1.4%)	43 (25.6%)	71 (19.7%)	110 (65.5%)	273 (75.6%)
Q2: Euthanasia in terminally ill patients with a short remaining life expectancy	6 (3.6%)	15 (4.2%)	9 (5.4%)	20 (5.5%)	5 (3.0%)	12 (3.3%)	45 (26.8%)	85 (23.5%)	103 (61.3%)	229 (63.4%)
Q3: PAS in patients with an incurable chronic illness, but not terminally ill	13 (7.7%	16 (4.4%)	20 (11.9%)	41 (11.4%)	11 (6.5%)	25 (6.9%)	55 (32.7%)	143 (39.6%)	69 (41.1%)	136 (37.7%)
Q4: PAS in the case of mental illness alone	23 (13.7%)	34 (9.4%)	28 (16.7%)	69 (19.1%)	15 (8.9%)	66 (18.3%)	56 (33.3%)	120 (33.2%)	46 (27.4%)	72 (19.9%)
Q5: PAS for people with tiredness of life who want to die but have no serious illness	31 (18.5%)	68 (18.8%)	34 (20.2%)	80 (22.2%)	20 (11.9%)	57 (15.8%)	44 (26.2%)	100 (27.7%)	39 (23.2%)	56 (15.5%)
Q6: The legalization of PAS may result in weak groups experiencing pressure to request aid in dying	17 (10.1%)	47 (13.0%)	54 (32.1%)	135 (37.4%)	33 (19.6%)	62 (17.2%)	47 (28.0%)	82 (22.7%)	17 (10.1%)	35 (9.7%)
Q7: Instead of allowing PAS, we should develop and expand the provision of palliative care to the dying	15 (8.9%)	27 (7.5%)	52 (31.0%)	83 (23.0%)	58 (34.5%)	149 (41.3%)	15 (8.9%)	49 (13.6%)	28 (16.7%)	53 (14.7%)
Q8: Treatment limitation can sometimes be the right decision, to avoid a distressing prolongation of the dying process	3 (1.8%)	5 (1.4%)	1 (0.6%)	11 (3.0%)	11 (6.5%)	10 (2.8%)	61 (36.3%)	108 (29.9%)	92 (54.8%)	227 (62.9%)
Q9: I support the decision that PAS is now allowed in Germany	9 (5.4%)	8 (2.2%)	6 (3.6%)	7 (1.9%)	8 (4.8%)	16 (4.4%)	45 (26.8%)	67 (18.6%)	100 (59.5)	263 (72.9%)
Q10: I am concerned that right to die organizations are financially enriching themselves	41 (24.4%)	65 (18.0%)	48 (28.6%)	149 (41.3%)	30 (17.9%)	69 (19.1%)	34 (20.2%)	59 (16.3%)	15 (8.9%)	19 (5.3%)
Q11: I would make use of PAS if I were suffering from a serious physical illness	9 (5.4%)	14 (3.9%)	16 (9.5%)	33 (9.1%)	35 (20.8%)	47 (13.0%)	38 (22.6%)	120 (33.2%)	70 (41.7%)	147 (40.7%)
Q12: I can generally understand why people would want to die through PAS	3 (1.8%)	0 (0%)	1 (0.6%)	1 (0.3%)	2 (1.2%)	5 (1.4%)	43 (25.6%)	70 (19.4%)	119 (70.8%)	285 (78.9%)
Q13: I am concerned that people may choose PAS too quickly and, for example, refuse palliative care	16 (9.5%)	30 (8.3%)	63 (37.5%)	142 (39.3%)	20 (11.9%)	52 (14.4%)	52 (31.0%)	109 (30.2%)	17 (10.1%)	28 (7.8%)
Q14: I cannot understand the wish for PAS at all	127 (75.6%)	305 (84.5%)	29 (17.3%)	48 (13.3%)	6 (3.6%)	7 (1.9%)	3 (1.8%)	1 (0.3%)	3 (1.8%)	0 (0%)
Q15: I think doctors in Germany should inform patients about the possibility of PAS as an alternative to treatment options	8 (4.8%)	17 (4.7%)	15 (8.9%)	47 (13.0%)	20 (11.9%)	39 (10.8%)	69 (41.1%)	123 (34.1%)	56 (33.3%)	135 (37.4%)
Q16: I would make use of PAS if I were suffering from a serious mental illness	21 (12.5%)	32 (8.9%)	28 (16.7%)	77 (21.3%)	43 (25.6%)	103 (28.5%)	35 (20.8%)	85 (23.5%)	41 (24.4%)	64 (17.7%)

*SL* Suicide Loss, *NSL* No Suicide Loss

Participants who had not experienced a loss by suicide had a significantly more liberal attitude towards the legalization of PAS in Germany than people who had experienced a suicide loss, *t*(265.32) = 2.78, *p* = 0.006., with an effect of *d* = 0.27. Individuals who had not been bereaved by suicide were also significantly more likely to understand why someone might choose PAS, *t*(237.04) = 2.30, *p* = 0.022., with a small effect of *d* = 0.23. Survivors of suicide loss were significantly less understanding of the general wish for PAS, *t*(218.76) = -2.88, *p* = 0.004., with an effect of *d* = 0.30. However, it should be noted that the effect is minimal and both groups have almost identical mean values. We could not find any differences between the groups regarding other attitudes towards PAS, such as PAS being allowed for individuals with a mental health disorder. All results are shown in Table 3.

**Table 3:** Differences in attitudes towards PAS and voluntary euthanasia between individuals with (*n* = 168) and without suicide loss (*n* = 361)

	**Suicide Loss**	**No Suicide Loss**	***t***	**df**	***p***	***d***
	***M (SD)***	***M (SD)***				
Q1: PAS should be permitted in terminally ill patients with a short remaining life expectancy	4.48 (.92)	4.67 (.71)	2.38	261.93	0.18	0.23
Q2: Euthanasia should be permitted in terminally ill patients with a short remaining life expectancy	4.37 (1.02)	4.37 (1.06)	-.04	527	.972	0.00
Q3: PAS should be permitted in patients with an incurable chronic illness, but not terminally ill	3.87 (1.28)	3.95 (1.14)	.63	294.13	.532	0.07
Q4: PAS should be permitted in the case of mental illness alone	3.44 (1.40)	3.35 (1.26)	-.70	296.31	.484	0.07
Q5: PAS should be permitted for people with tiredness of life who want to die but have no serious illness	3.15 (1.46)	2.99 (1.37)	-1.27	527	.205	0.11
Q6: The legalization of PAS may result in weak groups experiencing pressure to request aid in dying	2.96 (1.91)	2.79 (1.21)	-1.53	527	.128	0.49
Q7: Instead of allowing PAS, we should develop and expand the provision of palliative care to the dying	2.93 (1.19)	3.05 (1.12)	1.08	527	.281	0.10
Q8: Treatment limitation can sometimes be the right decision, to avoid a distressing prolongation of the dying process	4.42 (.79)	4.50 (.81)	1.09	527	.276	0.10
Q9: I support the decision that PAS is now allowed in Germany	4.32 (1.08)	4.58 (.85)	2.78	265.32	**.006**	0.27
Q10: I am concerned that PAS organizations are financially enriching themselves	2.61 (1.30)	2.50 (1.12)	-1.00	287.52	.338	0.09
Q11: I would make use of PAS if I were suffering from a serious physical illness	3.86 (1.22)	3.98 (1.12)	1.09	303.25	.277	0.10
Q12: I can generally understand why people would want to die through PAS	4.63 (.71)	4.77 (.47)	2.30	237.04	**.022**	0.23
Q13: I am concerned that people may choose PAS too quickly and, for example, refuse palliative care	2.95 (1.22)	2.90 (1.15)	-.45	527	.656	0.04
Q14: I cannot understand the wish for PAS at all	1.37 (.79)	1.18 (.45)	-2.88	218.76	**.004**	0.30
Q15: I think doctors in Germany should inform patients about the possibility of PAS as an alternative to treatment options	3.89 (1.11)	3.86 (1.19)	-.26	527	.793	0.03
Q16: I would make use of PAS if I were suffering from a serious mental illness	3.28 (1.34)	3.20 (1.22)	-.66	299.84	.508	0.06

two-tailed significant

### Attitudes towards PAS and voluntary assisted dying and demographic factors

Firstly, we analyzed the differences in general attitudes (first five questions of NOBAS) between the age groups 18–29, 30–39, 40–49, 50–59, and 60 + years while controlling for suicide loss. Individuals in the oldest subgroup showed the most negative attitudes towards the legalization of voluntary euthanasia for terminally ill patients (see Table 4). There were no significant differences between the age groups for the other items. Further, the differences in attitudes among people with secondary, higher secondary, and higher education were analyzed, while controlling for suicide loss. People with the highest level of education showed a more negative attitude towards voluntary euthanasia than the other subgroups. We then analyzed the attitudes of participants of different religions. However, only individuals of no religious belief, Protestant, and Catholic were assessed, as the sample of other religions was too small for the analysis. Participants of Catholic belief showed significantly more negative attitudes towards the legalization of PAS and voluntary euthanasia for all five items. In contrast, those with no religious beliefs showed significantly more liberal attitudes. However, it should also be noted here that the differences in mean values are minimal and no strong differences were found between the groups.

**Table 4:** Comparison of attitudes considering socio-demographic factors (*N* = 529)

Subgroups	Q1	Q2	Q3	Q4	Q5
Age					
18–29	4.62 (0.83)	4.36 (0.93)	3.90 (1.10)	3.36 (1.38)	2.76 (1.41)
30–39	4.72 (0.63)	4.50 (0.91)	3.99 (1.12)	3.38 (1.30)	3.21 (1.36)
40–49	4.62 (0.72)	4.45 (0.96)	3.94 (1.10)	3.43 (1.28)	2.99 (1.40)
50–59	4.48 (0.94)	4.25 (1.15)	3.83 (1.36)	3.27 (1.38)	3.02 (1.44)
60 +	4.53 (0.19)	3.98 (1.49)^a^	3.93 (1.30)	3.51 (1.33)	3.04 (1.38)
Education					
Secondary	4.64 (0.75)	4.56 (0.87)	3.86 (1.23)	3.39 (1.37)	3.02 (1.44)
Higher secondary	4.65 (0.78)	4.40 (1.02)	3.92 (1.18)	3.33 (1.30)	3.01 (1.42)
Higher	4.54 (0.82)	4.20 (1.17)^b^	3.97 (1.17)	3.44 (1.26)	3.08 (1.35)
Religion					
No religion	4.72 (0.64)	4.50 (0.92)	4.13 (1.06)	3.70 (1.16)	3.30 (1.35)
Protestant	4.55 (0.93)	4.33 (1.09)	3.80 (1.27)	3.20 (1.38)	2.86 (1.40)
Catholic	4.43 (0.86)^c^	4.09 (1.25)^c^	3.61 (1.27)^c^	2.86 (1.34)^c^	2.71 (1.42)^c^

Q1 = PAS for terminally ill patients, Q2 = Voluntary euthanasia for terminally ill patients, Q3 = PAS for patients with chronic illness, Q4 = PAS for people with mental health disorder, Q5 = PAS who are tired of life

^a^Significantly lowest mean of all age groups

^b^Significantly lowest mean of all education levels

^c^Significantly lowest means of all religious beliefs

### Opinions on PAS: Qualitative findings

The open question asking participants for their opinion on PAS was not a mandatory question, so a total of *n* = 239 participants completed the open response format. A total of four categories were identified: (1) autonomy and dignity, (2) impact on relatives and bereavement, (3) avoidance of violent deaths and suffering, and (4) PAS for mental health disorders and physical illness. All categories are described in more detail below, and it is also indicated whether the comment comes from a survivor of suicide loss (SL) or a person who has not lost someone to suicide (NSL). Not all participants completed the free text field, as this question was optional.

#### Autonomy and dignity

In total, *n* = 62 people mentioned a person’s autonomy and right to make decisions regarding their own death. Many participants also stated their own desire to die in a self-determined way. Making decisions regarding one’s own death and not having to wait for it to occur naturally was viewed as allowing one to die with dignity.

*“If a person is suffering so much, whether physically or mentally, then they should be able to decide whether they want to leave this world and also decide when.”* (SL)

*“In my opinion, being able to leave the world with dignity and autonomy is a human right.”* (NSL)

However, many participants also emphasized the importance of ensuring that the wish is autonomous and stable, while highlighting the need for multiple consultations with doctors or right-to-die organizations to avoid spontaneous decisions for PAS. Although some participants emphasized the importance of autonomy, concerns that the wish could be expressed at short notice and that other treatment options would not be attempted first were also reported.

*“I think PAS makes complete sense if it really is out of the question that the person wants to continue living. It should not be a spontaneous decision.”* (SL)

*“It may be chosen too quickly. Hopelessness and the desire to die are not always permanent; it is an illness. So where do you draw the line? “ *(SL)

PAS can also be a way of preventing an undignified death associated with great pain. This also considers that palliative care sometimes reaches its limits and not everyone can be cared for as painlessly as possible at the end of life. Other participants reported concerns that palliative care services might not be used to their full extent.

*“I work in a hospice, and in rare cases even palliative medicine reaches its limits, so I think everyone should be allowed to decide independently when a situation is no longer bearable.”* (NSL)

“*However, I also think it is important to offer more palliative care and to expand it so that an assisted suicide does not have to be necessary and the only way out. I think consciously living through the dying phase can also be very important*.” (NSL)

Some participants also stated that the option of PAS is associated with feelings of relief, in that one could make decisions regarding their own death, and limit suffering, in the event of an illness.

*“If I have missed my own last chance, I find it comforting to know that I could get help when the palliative options have been exhausted.”* (SL)

#### Impact on relatives and bereavement

The impact of PAS on relatives was mentioned almost exclusively by survivors of suicide loss. A total of *n* = 32 survivors spoke about their own experience and what consequences PAS could have for those close to the deceased person.

*“Suicide is the cruelest way to die and means incomprehensible suffering for relatives. Assisted dying opens the possibility of saying goodbye and may not leave relatives with so many unanswered questions and horror images.”* (SL)

*“I think because I know the pain of suicide too well, it is very difficult to think about the topic in a neutral way on an emotional level (…), but if someone around me would decide to do it I would absolutely reject it.”* (SL)

The lack of an opportunity to say goodbye is usually perceived as highly distressing for the bereaved. PAS could give those affected the opportunity to say goodbye, prepare for the death, and talk things through. Several bereaved participants stated that this would have helped them in the grieving process, as they did not have the opportunity to say goodbye to the person after the suicide took place.

*“It would be better if you could say goodbye and hug the person (…). Instead of dying in secret, in pain, and alone, relatives could hold the person’s hand and support them. I wish I could have spoken to my sister one last time.”* (SL)

*“I would have loved to have been able to say goodbye and hold her hand in the last few minutes.”* (SL)

Participants stated that they would have liked to have accompanied their loved ones— to have been there— so that the person did not have to die alone. Many reported that the thought of the person dying while feeling lonely to be highly distressing. PAS could give relatives the opportunity to be there for the dying person.

*“I am convinced that no one should leave the world alone. Everyone should be able to feel the comfort and security of a familiar person.”* (SL)

*“I think accompanying the person is very very important. For the person who is dying but also for the bereaved. I would have liked to hold my husband’s hand to show him that I am here and that he is not alone in this moment.”* (SL)

After the loss, survivors of suicide loss are also often confronted with feelings of guilt and responsibility for the suicide. Questions regarding the reason behind the suicide are often also present. Participants stated that these grief symptoms might not occur in the event of a loss through PAS.

*“The grief for my partner would certainly have been the same, but it would not have been accompanied by images of horror and feelings of guilt. That’s what makes grieving after suicide so difficult.”* (SL)

However, some bereaved participants opposed the legalization of PAS due to their own experiences. These individuals view assisted dying critically, expressing the high burden it places on relatives.

*“I can’t imagine PAS at all, I’ve lost two people to suicide in the last year, it’s hard as a relative.”* (SL)

*“A difficult topic from my point of view as a bereaved person whose life has changed so much after the suicides of my husband and my brother. The pain and helplessness within the family is so significant that I find it hard to have an opinion on that.”* (SL)

### Avoidance of violent deaths and suffering

Both bereaved individuals and those without a suicide loss considered PAS to be a less violent death than suicide, and could be associated with less pain for both the deceased person as well as the bereaved.

*“I would have wished for a less agonizing death for him. He drove his car into a tree.”* (SL)

*“In my opinion, a death through PAS would have been more humane and dignified than the agonizing and slow death by poisoning that she chose.”* (SL)

Dying by PAS could also mean a gentler death than through suicide and might not entail the risk of long-term suffering. Some survivors also mentioned that the deceased person was still in a coma after the suicide and that they found this to be an additionally stressful experience.

*“My mother was in a coma for three weeks after her suicide in an intensive care unit. That was the worst time for her and us.”* (SL)

*“I would like to have this option instead of the agonizing alternatives, which also make it worse for the relatives, like slitting your wrist. It’s about dying with dignity and not through a painful and brutal suicide.”* (NSL)

### PAS for mental health disorders and physical illnesses

It became evident that even among the participants, regardless of whether they had experienced a suicide loss or not, there was disagreement regarding PAS in cases of mental health disorders. The responses of *n* = 34 participants were related to this topic. While some individuals emphasized the relevance of self-determination, regardless of the presence of an underlying illness, others limited self-determination to only in the case of incurable physical illnesses.

*“Even if it is very difficult, the possibility of assisted dying should depend on personal suffering and not on the type of illness or impairment.“* (SL)

Some participants supported the legalization of PAS, but only in the case of physical illnesses. Some were concerned that the wish to die in individuals with mental health disorders may not be stable and due to a temporary crisis, which could recede again with therapeutic support. Some also reported their own suicidal thoughts during mental crises and that they could not rationally decide whether they wanted to live or die at that time.

*“In the case of mental health disorders, I find it somewhat difficult to decide. I find it hard to grasp whether you can really make the decision with a clear mind and full consciousness.”* (SL)

*“I find it particularly hard in the field of mental health, because there are many suicide attempts and people are very happy they did survive.”* (NSL)

In terms of mental health disorders, participants tended to emphasize the need for rapid suicide prevention support services and an expansion of therapeutic support. Some suggested developing separate regulations for PAS and voluntary euthanasia for people with mental health disorders. These suggestions highlighted the need for individuals to receive therapeutic support for a certain period before PAS is made possible. Others recommended monitoring individuals to assess the stability of their wishes over time.

*“I take a different view of mental health disorders, where treatment options should first and foremost be expanded. And, above all, no patient at risk of suicide should have to spend months looking for therapy.”* (SL)

## Discussion

The aim of the study was to provide an overview of attitudes towards assisted dying in individuals with and without suicide loss and to analyze the differences between the two groups, while also considering relevant socio-demographic factors. While previous research has examined the attitudes towards PAS across various population groups, so far, the own experience of suicide loss has not yet been included as a research perspective.

Overall, participants showed a positive attitude towards physician-assisted suicide and voluntary euthanasia for people with a terminal illness and a positive attitude towards the legalization of PAS in Germany, regardless of their experience of suicide loss. Regarding access for people without a physical illness, participants were rather divided in their attitudes. These results are in line with studies on attitudes in the general population [4, 24]. Participants could also understand why people make use of PAS and were less concerned that right-to-die organizations could profit from it. Only few small differences between people with and without loss by suicide experiences were found. Participants without loss by suicide experiences showed more liberal attitudes towards legalization in Germany and were more likely to understand the wish for assisted dying. Survivors of suicide loss showed significantly less understanding of the wish for PAS. However, the difference is minimal and only statistically and not clinically and ethically significant. It is possible that larger differences between groups could be found if the sample were bigger or other factors were considered.

It is therefore not possible to conclusively conclude whether attitudes towards PAS are shaped by one’s own experience of loss. Further research is urgently needed to shed more light on this topic. Loss by suicide is associated with severe grief symptoms in the bereaved [14, 25], as well as feelings of guilt and questions regarding the reasons behind the suicide. The pain of loss can lead to the bereaved being less liberal towards PAS due to the known consequences of death, as they know first-hand how devastating it can be and what effects it has on relatives. This population group may also be less sympathetic to the decision of PAS, as they might not understand how a person could choose an unnatural death and leave their relatives behind. However, some participants who experienced a suicide loss gave the impression that they saw assisted suicide as an alternative to (non-assisted) suicide. It is possible that this assumption influences the attitude towards assisted suicide and that participants perceive it more positively. Even if the questionnaire did not imply this, some suicide bereaved participants may have perceived it as such, which could in turn shape their attitudes.

This point is also reflected in the qualitative results, in which participants were asked to share their opinions on PAS. Survivors of suicide loss were particularly concerned about the impact on relatives in the event of assisted dying. Precisely because they have experienced a suicide loss, as well as the associated grief, these participants found the decision for PAS difficult to imagine. However, bereaved participants also emphasized the opportunity to say goodbye to the dying person and to be involved in the dying process, which might have a positive impact on bereavement. In their systematic review, [18] found that, in most studies, relatives who lost someone through PAS showed similar or even lower levels of grief and psychopathological outcomes than those who lost someone through a natural death. Against this background, it seems particularly helpful for individuals to be involved in the end-of-life decision-making process and to be able to prepare for the death. In another study, Snijdewind et al. [1] interviewed individuals who lost a partner through suicide or through physician-assisted dying due to a mental health disorder. Participants who lost their partner through physician-assisted dying showed lower grief reactions. Reasons for this could be that partners were involved in the decision-making process, were able to say goodbye and knew that the deceased chose death of their own free will. However, Wagner and Maercker [26] found increased depression and PTSD symptoms in relatives who were themselves present when the person died. Accompanying the dying process can, therefore, also have a negative impact on the mental health of relatives. However, the most frequently mentioned aspect among all participants was autonomy, which is consistent with several other studies on attitudes towards PAS and voluntary euthanasia [5, 6, 27]. Regardless of any existing experience of loss, autonomy seemed to be the most prevalent variable among the participants.

Lastly, we also found some small differences between groups of different age, education, and religion in some items. Older participants and those with a higher level of education were less in favor of voluntary euthanasia. People with no religious beliefs showed the most liberal attitudes towards PAS and voluntary euthanasia. Here, we also found statistically significant differences, but no major differences between the groups can be seen when considering the mean values. Nevertheless, these results are in line with previous studies that looked at attitudes towards PAS in different socio-demographic subgroups [2, 3, 28].

To the best of our knowledge, this study is the first to analyze attitudes towards PAS and voluntary euthanasia while considering individual’s own experience of suicide loss. We have a sufficiently large and heterogeneous sample in terms of age, level of education, religious belief, and experience of loss. The qualitative data also allowed us to examine a broad range of opinions, although the results should be interpreted against the background of some limitations. Our sample consisted of over 90% female participants, which means that generalizability is limited. One reason could be that we have mainly recruited via our social media channels, where significantly more women follow us than men, possibly due to a greater interest in psychology and mental health. The results therefore largely reflect the opinions of women. The cross-sectional design also does not allow any inferences about causality. Although the measurement instruments were used in a previous study, these have not been validated and can only be analyzed at item level. Conducting multiple tests can elevate the risk of random significant results and increase the likelihood of false-positive results. Since our analysis was performed at item level, no correction was applied. However, a questionnaire with the possibility of calculating subscales would be recommended for further analyses. The study was also not preregistered. We also had a high dropout rate at the beginning of the questionnaire. Most individuals didn’t even start the questionnaire after reading the first page. Nonetheless, the dropout rate may lead to a selection bias and can influence the generalizability of our findings. Although the two groups did not differ significantly in the socio-demographic data that we collected, it cannot be excluded that they differed in terms of other factors. For example, we did not collect data on political orientation, ethnic or cultural background, which could have an influence on opinions on PAS.

## Conclusions

This study provides the first important insights into the attitudes of survivors of suicide loss and the general public regarding the regulations on PAS and voluntary euthanasia in Germany. So far, little is known about how the general population in Germany sees the new legal situation regarding PAS and how one’s own experience of suicide loss shape their attitudes. While the present results provide an overview of possible concerns and fears of individuals regarding PAS and voluntary euthanasia, they also highlight how complex and multi-layered this topic can be. Future research should address this topic in more detail, exploring a broader range of aspects when surveying attitudes while also including individuals who lost someone through PAS.

## Supplementary Information

Supplementary Material 1.

## Data Availability

The data can be requested from the first author.
