# Supplementary material for: What do suicide loss survivors think of physician-assisted suicide: a comparative analysis of suicide loss survivors and the general population in Germany
Source: BMC Med Ethics. 2024 Sep 19;25:98. doi: 10.1186/s12910-024-01099-9 (PMC11412012; doi:10.1186/s12910-024-01099-9)
Supplement: Supplementary file 1 — Supplementary Material 1. [file 12910_2024_1099_MOESM1_ESM.docx]

**Supplementary Material**

***Table S1.*** *Questionnaire on the legalisation of assisted suicide in Germany*

|  | Strongly disagree | Somewhat disagree | Neither agree or disagree | Somewhat agree | Strongly agree |
| --- | --- | --- | --- | --- | --- |
| 1. I am in favour of the decision that assisted suicide is now permitted in Germany. | 🞏 | 🞏 | 🞏 | 🞏 | 🞏 |
| 1. I am concerned that the right-to-die organizations are getting financially rich from assisted suicides. | 🞏 | 🞏 | 🞏 | 🞏 | 🞏 |
| 1. I would make use of assisted suicide if I were suffering from a serious physical illness. | 🞏 | 🞏 | 🞏 | 🞏 | 🞏 |
| 1. I can generally understand that people want to make use of assisted suicide. | 🞏 | 🞏 | 🞏 | 🞏 | 🞏 |
| 1. I am concerned that people could decide in favour of assisted suicide too quickly and, for example, refuse palliative care. | 🞏 | 🞏 | 🞏 | 🞏 | 🞏 |
| 1. I cannot understand the wish for assisted suicide at all. | 🞏 | 🞏 | 🞏 | 🞏 | 🞏 |
| 1. I think doctors in Germany should inform patients about the possibility of assisted suicide as an alternative to other treatment options. | 🞏 | 🞏 | 🞏 | 🞏 | 🞏 |
| 1. I would make use of assisted suicide if I were suffering from a serious mental health disorder. | 🞏 | 🞏 | 🞏 | 🞏 | 🞏 |
